# Global monthly gridded atmospheric carbon dioxide concentrations under the historical and future scenarios

**DOI:** 10.1038/s41597-022-01196-7

**Published:** 2022-03-11

**Authors:** Wei Cheng, Li Dan, Xiangzheng Deng, Jinming Feng, Yongli Wang, Jing Peng, Jing Tian, Wei Qi, Zhu Liu, Xinqi Zheng, Demin Zhou, Sijian Jiang, Haipeng Zhao, Xiaoyu Wang

**Affiliations:** 1 Institute of Geographic Sciences and Natural Resources Research, Chinese Academy of Sciences, Beijing, 100101 China; 2 Key Laboratory of Land Surface Pattern and Simulation, Chinese Academy of Sciences, Beijing, 100101 China; 3 Key Lab of Regional Climate-Environment for Temperate East Asia, Institute of Atmospheric Physics, Chinese Academy of Sciences, Beijing, 100029 China; 4 University of Chinese Academy of Sciences, Beijing, 100049 China; 5 Institute of Tibetan Plateau and Polar Meteorology, Chinese Academy of Meteorological Sciences, Beijing, 100081 China; 6 Department of Earth System Science, Tsinghua University, Beijing, 100084 China; 7 School of Information Engineering, China University of Geosciences, Beijing, 100083 China; 8 Technology Innovation Center of Territory Spatial Big-data, MNR of China, Beijing, 100036 China; 9 College of Resource, Environment and Tourism, Capital Normal University, Beijing, 100048 China; 10 Key Laboratory of 3D Information Acquisition and Application of Ministry, Capital Normal University, Beijing, 100048 China

**Keywords:** Atmospheric chemistry, Atmospheric dynamics

## Abstract

Increases in atmospheric carbon dioxide (CO_2_) concentrations is the main driver of global warming due to fossil fuel combustion. Satellite observations provide continuous global CO_2_ retrieval products, that reveal the nonuniform distributions of atmospheric CO_2_ concentrations. However, climate simulation studies are almost based on a globally uniform mean or latitudinally resolved CO_2_ concentrations assumption. In this study, we reconstructed the historical global monthly distributions of atmospheric CO_2_ concentrations with 1° resolution from 1850 to 2013 which are based on the historical monthly and latitudinally resolved CO_2_ concentrations accounting longitudinal features retrieved from fossil-fuel CO_2_ emissions from Carbon Dioxide Information Analysis Center. And the spatial distributions of nonuniform CO_2_ under Shared Socio-economic Pathways and Representative Concentration Pathways scenarios were generated based on the spatial, seasonal and interannual scales of the current CO_2_ concentrations from 2015 to 2150. Including the heterogenous CO_2_ distributions could enhance the realism of global climate modeling, to better anticipate the potential socio-economic implications, adaptation practices, and mitigation of climate change.

## Abstract (summary)

**Table:** 

Measurement(s)	atmospheric carbon dioxide concentrations
Technology Type(s)	satellite observations • spatial statistical analysis

## Background & Summary

Recent satellite retrievals provide a continuous global spatial products of both column CO_2_, e.g., from the Chinese Global Carbon Dioxide Monitoring Scientific Experimental Satellite (TanSat), the Orbiting Carbon Observatory-2 (OCO-2) and the Greenhouse Gases Observing Satellite (GOSAT); and also mid-tropospheric CO_2_, e.g., the atmospheric infrared sounder (AIRS), those reveal the nonuniform distributions of mid-tropospheric CO_2_ concentrations^1–6^. The satellite-derived distributions of tropospheric CO_2_ are generally consistent with each other, though some regional discrepancies between the satellite products have been attributed to lack of independent reference observations constraints^5,7^. The areas with low atmospheric CO_2_ concentrations are in the high latitudes and the lack of any large CO_2_ emissions areas^1^. The areas with relatively high CO_2_ concentrations (30°S-60°N) are formed due to high CO_2_ emissions from ground sources, and the horizontal and vertical movements of winds^1,8^. These satellite CO_2_ concentrations retrievals provide a potential opportunity to investigate atmospheric CO_2_ variability at the planetary scale.

Few climate simulation studies have been based on a globally non-uniform mean CO_2_ distribution patterns^9–11^. Those produce a bias reduction in estimated mean temperatures, and consequently some understanding of the response of Earth’s system to the actual nonuniform CO_2_ concentrations. In the Beijing Normal University Earth System Model (BNU-ESM), the inhomogeneous CO_2_ simulations are driven by annual CO_2_ concentrations with spatial and seasonal changes derived from satellite observation^10^. While in the Community Earth System Model (CESM), spatially inhomogeneous CO_2_ runs use prescribed gridded national-level monthly or annual CO_2_ emissions weighted by the grid’s population density^9,11^. Both BNU-ESM and CESM simulations with spatially inhomogeneous CO_2_ reproduce the progressive increases in temperature with better agreement with spatially distributed global surface air temperature observations than using spatially homogeneous simulations^10,11^. The heterogenous CO_2_ distributions could enhance the realism of global climate modeling.

Climate modeling taking into account the CO_2_ distribution could address some of the known biases in temperature in the control simulations^11^. Including the heterogenous CO_2_ distribution could enhance the realism of global climate modeling. Using BNU-ESM, global mean surface air temperature is in the inhomogeneous CO_2_ simulations is approximately 0.3 °C lower than that in spatially uniform runs over the period 1986–2005, reducing the warming bias seen in the uniform runs compared with the HadCRUT4 observations^10^. In CESM, spatially homogeneous CO_2_ simulations overestimated climate warming over the Arctic, tropical Pacific, while underestimated warming in the mid-latitudes, over most land areas^9^. The inhomogeneous runs simulated by CESM during 1950–2000 produces lower temperatures at both poles than the homogeneous runs, by up to 1.5 °C including statistically significant cooling over the Barents Sea area^11^.

The surface air temperature responses to spatially inhomogeneous atmospheric CO_2_ concentrations are mainly controlled by changes in large scale atmospheric circulations, e.g., the Hadley cell, westerly jet, Arctic Oscillation and Rossby waves^8–11^. Local surface air temperature anomalies under nonuniform CO_2_ simulations are affected by the CO_2_ physiological response over vegetated areas. The land plants adjust to changes in atmospheric CO_2_ by altering their stomatal conductance, which consequently affects the water evapotranspiration from plant leaf to atmosphere^12^. This affects environmental temperature through evaporative cooling, and the evaporated moisture alters the air humidity and influences low cloud amounts by the water vapor diffusion, which is especially obvious in summer when the plants grow vigorously. In the polar areas, the degree of warming amplification depends strongly on the locally distribution of CO_2_ radiative forcing, specifically through positive local lapse-rate feedback, with ice-albedo and Planck feedbacks playing subsidiary roles, also suggesting that inhomogeneous spatial distributions of CO_2_ concentrations is consistent with significant climatic effects^13^. In marine ecosystems, non-uniform atmospheric CO_2_ and temperature biases could affect the uptake and storage of CO_2_ in the ocean, which will change regional atmospheric CO_2_ concentrations, ocean pH, ocean oxygen concentrations and primary production^14^.

Existing studies with spatially homogeneous atmospheric CO_2_ concentrations may have underestimated the temperature gradient from mid-latitudes to high latitudes. Some atmospheric circulation patterns, e.g., the Hadley cell, westerly jet and Arctic Oscillation are theoretically related to the mid- to high-latitude temperature gradients, and are hence potentially incorrectly simulated^9^. Spatially homogeneous atmospheric CO_2_ simulations underestimate interannual variability in regional temperature and precipitation relative to the inhomogeneous simulations^9^ and so can result in underrating magnitudes and frequencies of extreme event such as droughts, heat waves, floods, and hurricanes^12^. The upper 3 m of Arctic permafrost holding twice as much carbon as the atmosphere is accelerating its thaw due to the intensification of Arctic warming, leading to Greenhouse gases release and accelerating global warming^15^. Biases of temperature from spatially uniform CO_2_ responses to ice-albedo-temperature feedbacks would lead to overestimated polar warming relative to inhomogeneously distributed CO_2_ in the historical period^13^.

However, climate simulation studies are almost based on a globally uniform mean CO_2_ or latitudinally resolved CO_2_ datasets for the historical and future scenarios in the Climate Model Intercomparison Project^16–19^. In the models including representation of the carbon cycle, the CMIP simulations can be driven by prescribed CO_2_ emissions accounting explicitly for fossil fuel combustion^19^. Feng *et al*.^20^ provided spatially distributed anthropogenic emissions historical data with annual resolution and future scenario data in 10-year intervals for CMIP6. There is near-real-time daily CO_2_ emission dataset monitoring the variations in CO_2_ emissions from fossil fuel combustion and cement production since January 1, 2019 at the national level^21^. Shan *et al*.^22^ constructed the time-series of CO_2_ emission inventories for China and its 30 provinces following the Intergovernmental Panel on Climate Change (IPCC) emissions accounting method with a territorial administrative scope. The other CMIP simulations can be driven by prescribed CO_2_ concentrations, which enables these more complex models to be evaluated fairly against those models without representation of carbon cycle processes^19^. Meinshausen *et al*.^17^ provided a prescribed global-mean greenhouse gases (GHGs) concentrations using atmospheric concentration observations and emissions estimates in the historical period (1750–2005) and using four different Integrated Assessment Models in the future scenario, with some models constraining internally generated fields of GHG concentrations to match those global-mean values. For CMIP6, Meinshausen *et al*.^18^ updated those global-mean and latitudinal monthly-resolved GHG concentration dataset in the historical period. In the future period, there are global annual mean GHG concentration dataset in some alternative scenarios of future emissions and land use changes produced with integrated assessment models^19^.

Here, we provide global monthly distributions of atmospheric CO_2_ concentrations with 1° resolution under historical (1850–2013) and future (2015–2150) scenarios in CMIP6, which have equal global annual mean values in the CMIP6 standard CO_2_ dataset. The monthly CO_2_ distributions dataset can be accessed by the Zenodo data repository^23^ (10.5281/zenodo.5021361). Climate modeling taking into account heterogenous CO_2_ distributions could reduce some of the known biases in the control simulations^9–11^, to better anticipate the potential socio-economic implications, adaptation practices, and mitigation of climate change.

## Methods

The historical CO_2_ concentrations follows CMIP6 monthly and latitudinally resolved CO_2_ concentrations accounting longitudinal features retrieved from fossil-fuel CO_2_ emissions from Carbon Dioxide Information Analysis Center. And the spatial distributions of CO_2_ under SSP-RCPs scenarios were generated based on the spatial, seasonal and interannual features of the current CO_2_ concentrations distributions.

### Historical CO_2_ concentrations spatial reconstruction

Since lack of observational evidence of both seasonality and latitudinal gradients of CO_2_ concentrations in pre-industrial times, CMIP6 project provides consolidated dataset of historical atmospheric concentrations of CO_2_ based on the Advanced Global Atmospheric Gases Experiment (AGAGE) and National Oceanic and Atmospheric Administration (NOAA) networks, firn and ice core data, and archived air data, and a large set of published studies for the earth system modeling experiments^18^. The dataset provides best-guess estimates of historical forcings with latitudinal and seasonal features (available at https://www.climatecollege.unimelb.edu.au/cmip6).

**Fig. 1:**
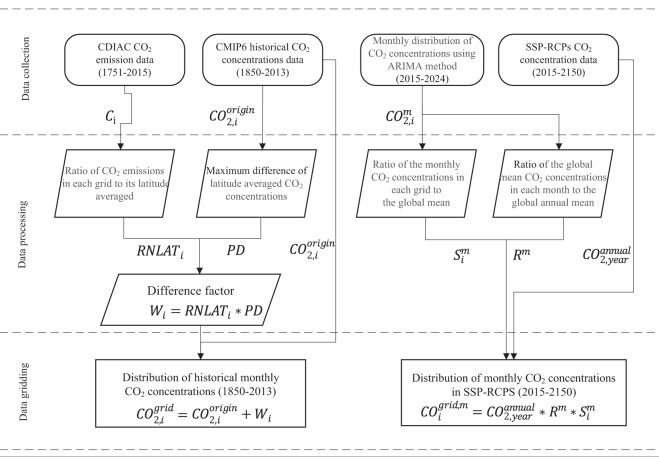
The processes for CO_2_ concentrations distributions reconstruction in the historical period and future scenarios.

The atmospheric CO_2_ concentrations from CMIP6 has only spatial distributions in latitude but not in longitude. We reconstructed the CMIP6 historical CO_2_ concentration data with global 1° resolution based on the fossil-fuel CO_2_ emissions data from Carbon Dioxide Information Analysis Centre (CDIAC). The CDIAC fossil-fuel CO_2_ emissions used here are based on fossil-fuel consumption estimates, which distributes spatially on a 1° latitude by 1° longitude grid from 1751 to 2013^24^. (available at https://cdiac.ess-dive.lbl.gov/trends/emis/meth_reg.html). However, there is no value of the CDIAC CO_2_ emissions over land without human activity and ocean, where CO_2_ emissions values are filled with the average values of their latitudes of CO_2_ emissions. The processed global carbon emissions data from CDIAC is used as features of CO_2_ distributions and seasonal cycle for downscaling historical atmospheric CO_2_ concentrations in each month (Fig. 1). The ratio of CDIAC CO_2_ emissions in each grid to its latitude averaged is calculated as:

1 $$RLA{T}_{i}={C}_{i}/{C}_{{\rm{LAT}}}$$

where *C*_*i*_ represents CO_2_ emission in each grid, and *C*_LAT_ is the corresponding latitude average CO_2_ emissions.

The ratio *RLAT*_*i*_ is normalized as,

2 $$RNLA{T}_{i}=\frac{RLA{T}_{i}-RLA{T}_{min}}{RLA{T}_{max}-RLA{T}_{min}}$$

where $RNLA{T}_{i}$ represents the normalized ratio *RLAT*_*i*_, $RLA{T}_{max}$ is the maximum value of *RLAT*_*i*_, and $RLA{T}_{min}$ is the minimum value of *RLAT*_*i*_.

The maximum difference of latitude averaged CO_2_ concentrations (*PD*) for CMIP6 data is calculated as,

3 $$PD=C{O}_{2,max}-C{O}_{2,min}$$

where $C{O}_{2,max}$ is the maximum latitude CO_2_ concentration, $C{O}_{2,min}$ represents the minimum latitude CO_2_ concentration.

The difference factor ${W}_{i}$ in each grid is calculated as,

4 $${W}_{i}=RNLA{T}_{i}\ast PD$$

The reconstructed CO_2_ concentrations $C{O}_{2,i}^{grid}$ equals to original CO_2_ concentrations and the difference factor in each grid, as

5 $$C{O}_{2,i}^{grid}=C{O}_{2,i}^{origin}+{W}_{i}$$

where $C{O}_{2,i}^{origin}$ is the CO_2_ concentrations in CMIP6.

### SSP-RCPs CO_2_ concentrations spatial reconstruction

In the future time period, CO_2_ concentration data for CMIP6 from 2015 were derived from the eight shared socioeconomic pathway (SSP) and representative concentration pathways (RCP) scenarios (Table 1) using the reduced-complexity climate–carbon-cycle model MAGICC7.0^25^. The five SSP scenarios SSP1-1.9, SSP1-2.6, SSP2-4.5, SSP3-7.0, and SSP5-8.5 that are used as priority scenarios highlighted in ScenarioMIP for the IPCC sixth assessment report^19^. The SSP1-1.9 and SSP1-2.6 are both in the “sustainability” SSP1 socio-economic pathway but with about 1.9 and 2.6 W m^−2^ radiative forcing level in 2100, reflecting ways for 1.5°C and 2°C targets under the Paris Agreement, respectively. The SSP2-4.5 follows “middle of the road” socio-economic pathway with a nominal 4.5 W m^−2^ radiative forcing level by 2100. The SSP3-7.0 is in the “regional rivalry” socio-economic pathway and a medium-high radiative forcing scenario. SSP5-8.5 marks the upper edge of the SSP scenario spectrum with a high reference scenario in a high fossil fuel development world throughout the 21st century. SSP5-3.4 follows SSP5-8.5, an unmitigated baseline scenario, through 2040, at which point aggressive mitigation is undertaken to rapidly reduce emissions to zero by about 2070 and to net negative levels thereafter. In addition, the SSP4-6.0 and SSP4-3.4 scenarios update the RCP6.0 pathway and fill a gap at the low end of the range of future forcing pathways, respectively. CMIP6 CO_2_ concentration data in each SSP-RCP scenario is available at https://esgf-node.llnl.gov/search/input4mips/.

**Table 1:** Summary for experimental scenarios designed in ScenarioMIP^19^.

Scenario name	SSP category	Forcing category	2100 forcing (W m^−2^)
SSP1-1.9	Sustainability	Low	1.9
SSP1-2.6	Sustainability	Low	2.6
SSP2-4.5	Middle of the road	Medium	4.5
SSP3-7.0	Regional rivalry	High	7.0
SSP4-3.4	Inequality	Low	3.4
SSP4-6.0	Inequality	Medium	6.0
SSP5-3.4	Fossil-fueled development	Low	3.4
SSP5-8.5	Fossil-fueled development	High	8.5

The global annual mean atmospheric CO_2_ in the CMIP6 future scenarios are interpolated temporally and spatially based on the features of CO_2_ distributions and seasonal cycle of the current monthly atmospheric CO_2_ concentrations distributions from 2015 to 2024 (the geotif2nc_2015_2024.nc file is contained within “Code.zip” archive accessed via the Zenodo data repository^23^ 10.5281/zenodo.5021361) simulated based on the monthly reconstructed historical CO_2_ concentrations using autoregressive integrated moving average (ARIMA) method^26,27^ (Fig. 1).

The ratio (${S}_{i}^{m}$) of the monthly CO_2_ concentrations in each grid to the global mean averaged during 2015–2024 is calculated as

6 $${S}_{i}^{m}=\frac{C{O}_{2,i}^{m}}{C{O}_{2,mean}^{m}}$$

where $C{O}_{2,i}^{m}$ is the monthly CO_2_ concentrations in each month *m* (*m* = 1, 2…12) and in each grid *i*. $C{O}_{2,mean}^{m}$ is the global mean CO_2_ concentrations in each month *m*.

The ratio (${R}^{m}$) of the global mean CO_2_ concentrations in each month to the global annual mean averaged during 2015–2024 is calculated as,

7 $${R}^{m}=\frac{C{O}_{2,mean}^{m}}{C{O}_{2,mean}^{annual}}$$

where $C{O}_{2,mean}^{annual}$ is the global annual mean CO_2_ concentration averaged during 2015–2024.

The CO_2_ concentrations distributions $C{O}_{i}^{grid,m}$ in each year are obtained by

8 $$C{O}_{i}^{grid,m}=C{O}_{2,year}^{annual}\times {R}^{m}\times {S}_{i}^{m}$$

where $C{O}_{2,year}^{annual}$ is the global annual mean CO_2_ concentrations in the CMIP6 future scenarios.

## Data Records

All atmospheric CO_2_ output grids can be accessed via the Zenodo data repository^23^ (10.5281/zenodo.5021361). The data records include 1 file Network Common Data Form (NetCDF) format for CO_2_ distributions in historical period named CO2_1deg_month_1850–2013.nc, and 8 files NetCDF format with the naming convention CO2_SSP{XYY}_2015_2150.nc, where X and YY are the shared socioeconomic pathway and radiative forcing level at 2100, respectively, for CO_2_ distributions in the future scenarios. Each NetCDF file includes 3 dimensions: time (month of the year expressed as days since the first day of 1850, n = 1968 and 1632 for the historical and the future, respectively); latitude (Degrees North of the equator [cell centres], n = 180); longitude (Degrees East of the Prime Meridian [cell centres], n = 360). Each NetCDF file contains a monthly variable representing mole fraction of carbon dioxide in air (variable name: values in the historical file and in the future scenario files) with the unit ppm and the 1° × 1° resolution. There are 127,526,400 and 105, 753,600 unique data points for the historical file and each future scenario file. All grids are bottom-left arranged with coordinates referenced to the prime meridian and the equator.

**Fig. 2:**
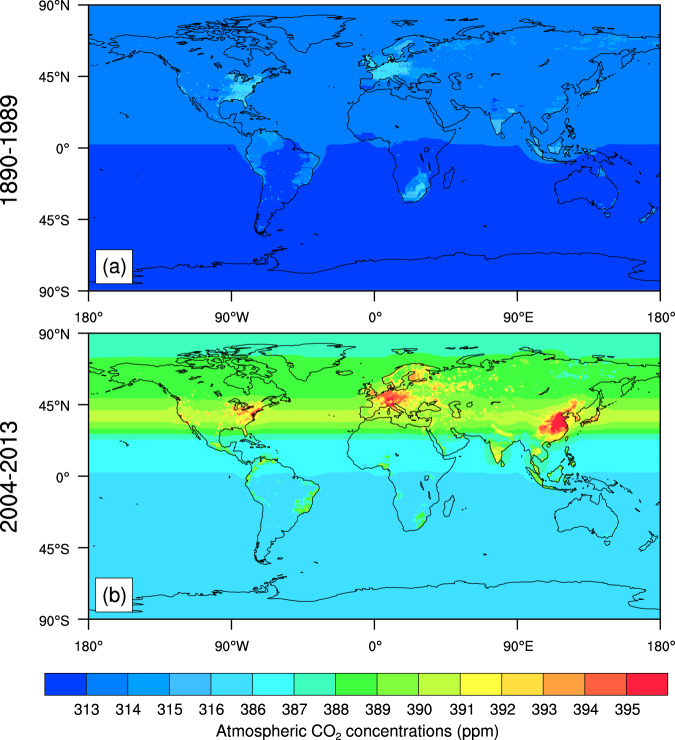
The maps of global historical atmospheric CO_2_ concentrations (ppm) averaged during 1890–1989 (Top) and averaged during 2004–2013 (Bottom).

**Fig. 3:**
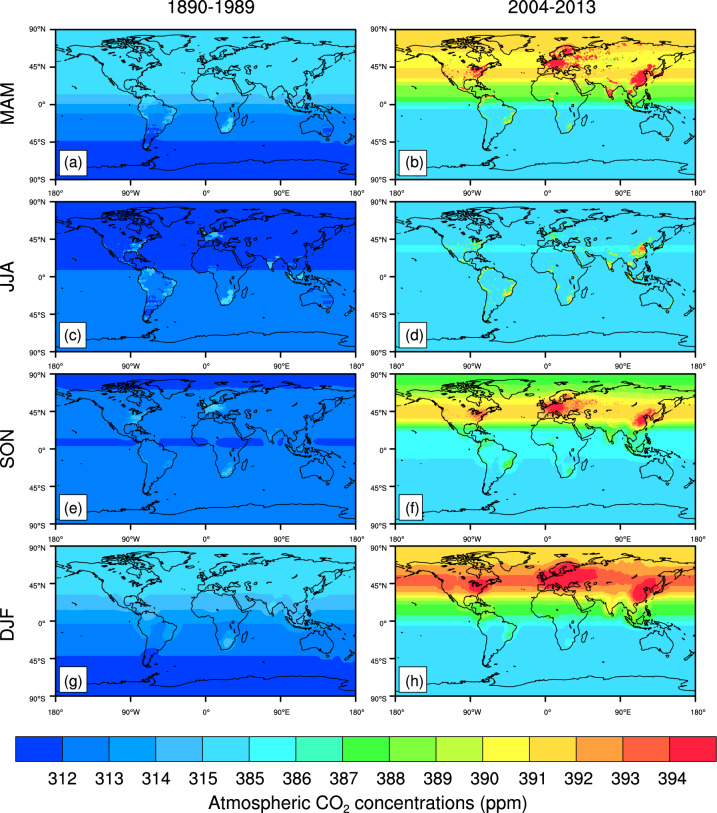
The maps of seasonal atmospheric CO_2_ concentrations (ppm) averaged during 1890–1989 (**a**) and averaged during 2004–2013 (**b**) in these March-April-May (MAM), June-July-August (JJA), September-October-November (SON), and December-January-February (DJF).

The spatial distributions of historical CO_2_ concentrations averaged during 1890–1989 shows that the high CO_2_ concentrations appears in the developed regions, e.g., Europe and Eastern part of the United States (Fig. 2). CO_2_ concentrations in the United Kingdom and the United States are 290.90 ppm and 287.83 ppm, respectively, during 1861–1880 (Table 2). During 2004–2013, the average CO_2_ concentrations in the United Kingdom and the United States increase to 391.65 ppm and 389.99 ppm, respectively (Table 2), which are associated with regional CO_2_ emissions. In addition, the CO_2_ concentration 391.16 ppm in China is slightly less than that in the United Kingdom, which is associated with the low CO_2_ concentrations in the west of China (Fig. 2). Fig. 3 shows the distributions of seasonal atmospheric CO_2_ concentrations (ppm) in these seasons March-April-May (MAM), June-July-August (JJA), September-October-November (SON), and December-January-February (DJF).

**Table 2:** Multi-year average atmospheric CO_2_ concentrations in various historical periods for some countries.

Regional CO_2_ concentrations (ppm)	1861–1880	1901–1920	1941–1960	1981–2000	2004–2013
Australia	287.83	299.66	313.07	353.11	384.32
Brazil	287.53	299.79	313.66	354.54	385.71
Canada	287.59	300.12	314.04	355.73	388.46
China	287.60	300.04	313.87	356.59	391.16
India	287.57	300.52	314.55	356.56	389.25
United States	287.83	300.8	314.61	356.25	389.99
United Kingdom	290.90	303.38	317.29	358.73	391.65

**Fig. 4:**
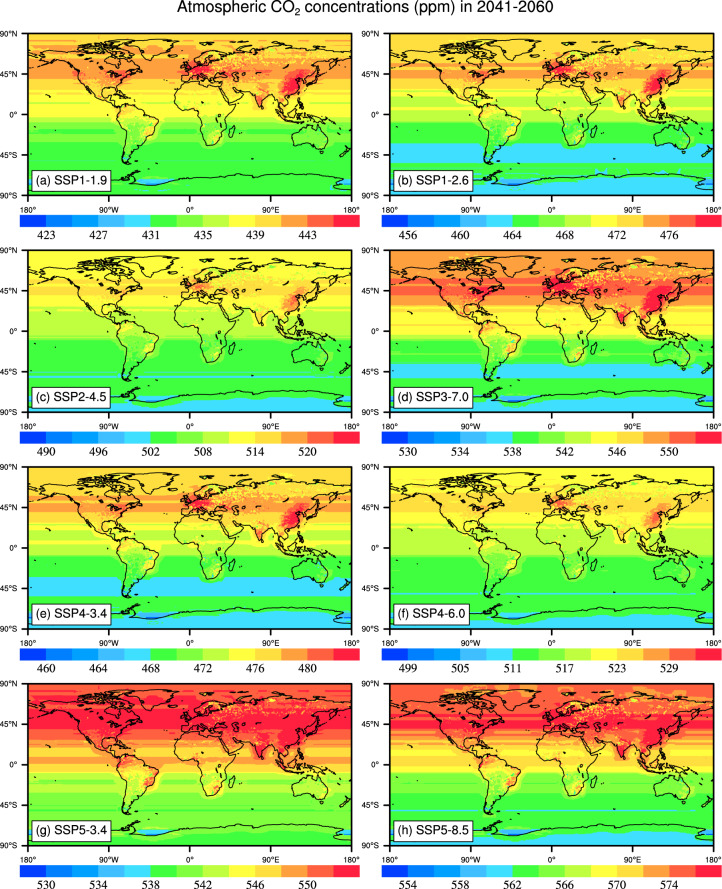
The maps of global atmospheric CO_2_ concentrations (ppm) averaged during 2041–2060 in the SSP1-1.9, SSP1-2.6, SSP2-4.5, SSP3-7.0, SSP4-3.4, SSP4-6.0, SSP5-3.4 and SSP5-8.5 scenarios. The period of 2041–2060 selected is for the average state in the middle of this century, the key time for carbon neutrality.

**Fig. 5:**
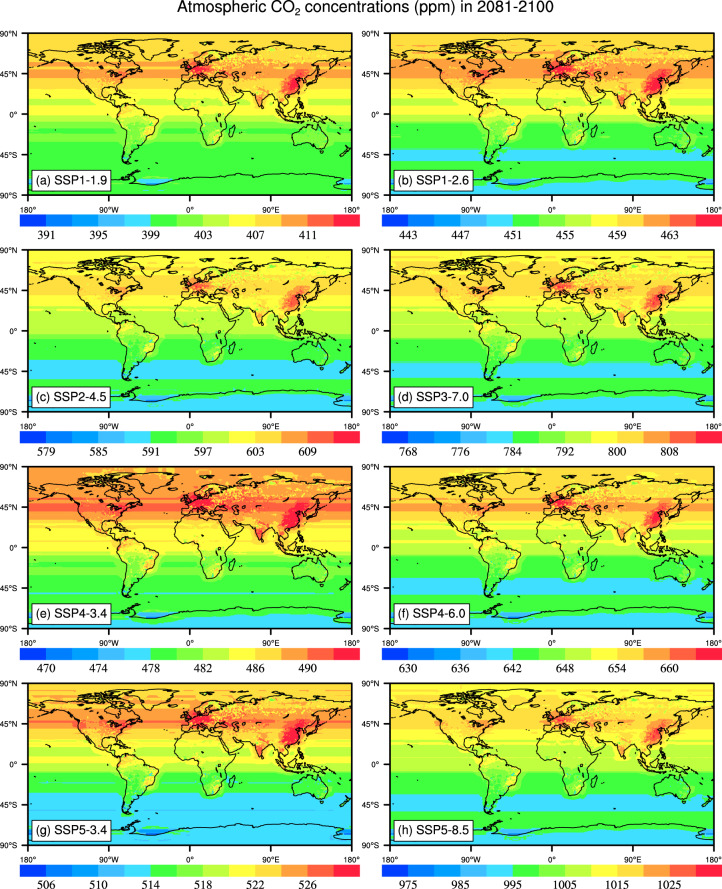
The maps of global atmospheric CO_2_ concentrations (ppm) averaged during 2081–2100 in the SSP1-1.9, SSP1-2.6, SSP2-4.5, SSP3-7.0, SSP4-3.4, SSP4-6.0, SSP5-3.4 and SSP5-8.5 scenarios. The period of 2081–2100 in the Fig. 5 chosen is for the average state at the end of this century.

CO2_SSP{XYY}_2015_2150.nc files are generated based on the eight SSP and RCP scenarios, including SSP1-1.9, SSP1-2.6, SSP2-4.5, SSP3-7.0, SSP4-3.4, SSP4-6.0, SSP5-3.4 and SSP5-8.5 which provide global distributions of CO_2_ concentrations under different socio-economic development pathway associated radiative forcing levels. In these eight scenarios, the average CO_2_ concentrations in the Northern Hemisphere (NH) is higher than that in the Southern Hemisphere (SH). High CO_2_ concentrations relative to the global average is mainly distributed in Europe, Eastern United States, and East Asia. Under each scenario, global CO_2_ concentrations averaged in 2041-2060 ranges 420–590 ppm, and the CO_2_ concentrations averaged during 2081–2100 is between 380–1030 ppm (Figs. 4, 5). Under SSP5-8.5, the average CO_2_ concentrations in China and the United Kingdom are 1020.70 ppm and 1021.51 ppm, respectively, during 2081–2100, while the CO_2_ concentration is 998.28 ppm in Australia (Table 3).

**Table 3:** Multi-year average atmospheric CO_2_ concentrations between 2081–2100 in some countries under future scenarios.

Regional CO_2_ concentrations during 2081–2100 (ppm)	SSP1-1.9	SSP1-2.6	SSP2-4.5	SSP3-7.0	SSP4-3.4	SSP4-6.0	SSP5-3.4	SSP5-8.5
Australia	401.47	452.88	592.52	786.85	480.57	644.23	514.99	998.28
Brazil	404.42	456.21	596.88	792.64	484.11	648.98	518.78	1005.63
Canada	408.62	460.95	603.07	800.87	489.13	655.71	524.16	1016.06
China	410.48	463.05	605.82	804.52	491.36	658.70	526.56	1020.70
India	408.62	460.95	603.08	800.88	489.13	655.72	524.17	1016.07
United States	409.14	461.54	603.85	801.90	489.76	656.55	524.84	1017.37
United Kingdom	410.81	463.42	606.31	805.17	491.75	659.23	526.98	1021.51

## Technical Validation

In this validation section, GOSAT surface CO_2_ concentrations and AIRS mid-tropospheric CO_2_ concentrations products were used for comparison with the reconstructed distributions of atmospheric CO_2_ concentrations. The GOSAT launched in January 2009 observing infrared light reflected and emitted from the earth’s surface and the atmosphere provides three-dimensional distributions of CO_2_ products calculated from the Level 4 A data product using a global atmospheric transport model from 2009^28–30^. The data product has a horizontal resolution of 2.5° × 2.5° and a time step of six hours. The satellite Aqua was launched in May 2002 and operates in a near polar sun-synchronous orbit, and its mission is to observe the global water and energy cycle, climate change trend, and response of the climate system to the increase in greenhouse gases^1,31^. It retrieves the global daily or monthly CO_2_ concentrations over land, ocean and polar regions^6^. AIRS mid-tropospheric CO_2_ concentrations product is retrieved using the Vanishing Partial Derivative method^32^, with the 90 km × 90 km spatial resolution covering 90°N–60°S. The AIRS CO_2_ retrieval product provides a continuous global nonuniform distributions of mid-tropospheric CO_2_ concentrations from 2003 to 2016.

**Fig. 6:**
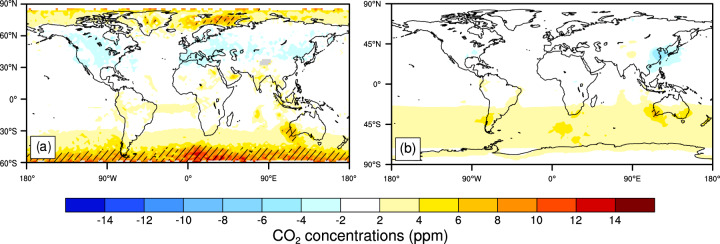
Changes in CO_2_ concentrations (ppm) between the reconstructed and AIRS (**a**) averaged during 2003–2016 (excluding 2014), and between the reconstructed and GOSAT (**b**) averaged during 2010–2018 (excluding 2014). The time periods selected are decided by data available. Hatched areas are regions where changes are statistically significant at the 5% level using the Student’s *t* test.

The multi-year mean reconstructed atmospheric CO_2_ concentrations are slightly higher than that of the AIRS mid-tropospheric CO_2_ concentrations product in the NH high latitudes and mid-latitudes of the SH, but lower in the mid-latitudes of the SH. In the 45°S-60°S latitude band, about 10 ppm (3%) increase in the reconstructed CO_2_ concentrations is statistically significant relative to the AIRS averaged during 2003–2016 (Fig. 6a). The reconstructed CO_2_ concentrations are about 4 ppm (1%) higher and lower than the GOSAT surface CO_2_ concentrations in the 30°S-60°S latitude band and in the East Asia and its adjacent sea areas, respectively, however, the biases are both not statistically significant at the 5% level using the Student’s *t* test (Fig. 6b).

**Fig. 7:**
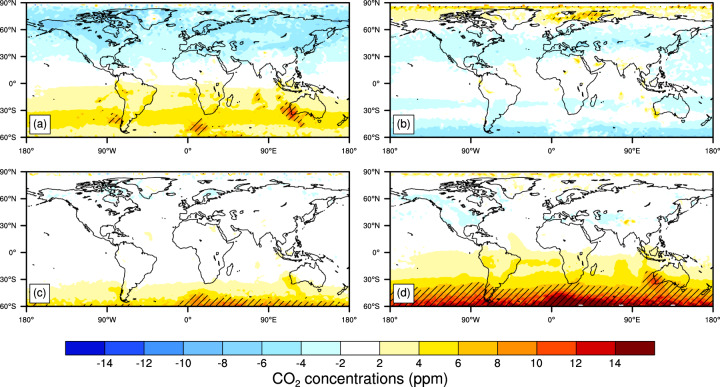
Changes in the seasonal (**a**, MAM; **b**, JJA; **c**, SON; **d**, DJF) CO_2_ concentrations (ppm) between the reconstructed and the AIRS product averaged during 2003–2016 (excluding 2014). The time period selected is decided by data available. Hatched areas are regions where changes are statistically significant at the 5% level using the Student’s *t* test.

Relative to the AIRS, there are some statistically significant seasonal overestimations of the reconstructed CO_2_ concentrations with over 12 ppm averaged during 2003–2016 mainly located in the 45°S–60°S latitude band in DJF (Fig. 7). In MAM, the reconstructed CO_2_ concentrations are 2-6 ppm lower in the NH and 2-6 ppm higher in the SH than that in the AIRS. In JJA, the reconstructed CO_2_ concentrations are 2-6 ppm lower at the latitude bands of 30°N–60°N, 15°S–30°S, and 45°S–60°S, and 2-6 ppm higher in the 60°N–90°N latitude band than that in the AIRS. In SON, the bias of the reconstructed CO_2_ concentrations is from −2 to 2 ppm in most regions of the world, except in 45°S-60°S latitude band relative to the AIRS.

**Fig. 8:**
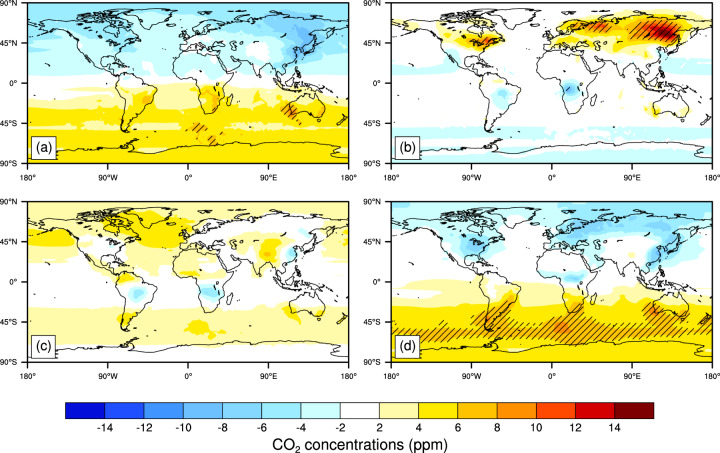
Changes in the seasonal (**a**, MAM; **b**, JJA; **c**, SON; **d**, DJF) CO_2_ concentrations (ppm) between the reconstructed and the GOSAT product averaged during 2010–2018 (excluding 2014). The time period selected is decided by data available. Hatched areas are regions where changes are statistically significant at the 5% level using the Student’s *t* test.

Relative to the GOSAT, there are some statistically significant seasonal overestimations of the reconstructed CO_2_ concentrations between 8 to 10 ppm averaged during 2010–2018 mainly at the 45°S-70°S latitude bands in DJF (Fig. 8). In JJA, the reconstructed CO_2_ concentrations are over 10 ppm higher than the GOSAT data in the Far eastern and North-western federal districts of Russia, and Eastern Canada. In MAM, the reconstructed CO_2_ concentrations are 2-8 ppm lower in the NH and 2-6 ppm higher in the SH than that in the GOSAT. In SON, the overestimations of the reconstructed CO_2_ concentrations are from 2 to 6 ppm, and the underestimations of the reconstructed CO_2_ concentrations is from −6 to −2 ppm in some areas of South America, South Africa and Eastern China relative to the GOSAT.

**Fig. 9:**
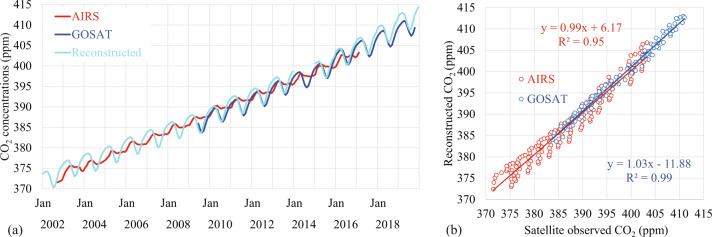
Monthly global mean time evolution of CO_2_ concentrations (ppm) for the AIRS (red, from Jan 2003 to Feb 2017), the GOSAT (blue, from Jun 2009 to oct 2017), and the reconstructed data (cyan) from 2003 to 2017 (**a**); and scatter plot between the monthly global mean reconstructed data, and the AIRS (red) and the GOSAT (blue), respectively averaged during 2010–2016 (**b**). The reconstructed CO_2_ data from 2010–2013 and 2015–2017 compared here is from the historical and the SSP5-8.5 reconstructions, respectively.

Compared with the GOSAT surface atmospheric CO_2_ concentrations, there is similar trend and seasonal cycles with the monthly global mean reconstructed CO_2_ concentrations (Fig. 9a). The seasonal cycle with high CO_2_ concentrations in MAM and low CO_2_ concentrations in JJA is closely related to the seasonal cycle of plant growth^33^. The monthly global mean AIRS mid-tropospheric CO_2_ concentrations have a similar trend and the peak feature of each seasonal cycle with the reconstructed and GOSAT CO_2_ concentrations, but the valley feature of seasonal cycles, which is associated with the transport of atmospheric CO_2_ and less impacts from plant CO_2_ absorption^34,35^. The R-squared correlation (R^2^) is 0.95 between the monthly global mean reconstructed CO_2_ and the AIRS CO_2_ product, and the R^2^ between the reconstructed and the GOSAT product is 0.99 (Fig. 9b).

**Fig. 10:**
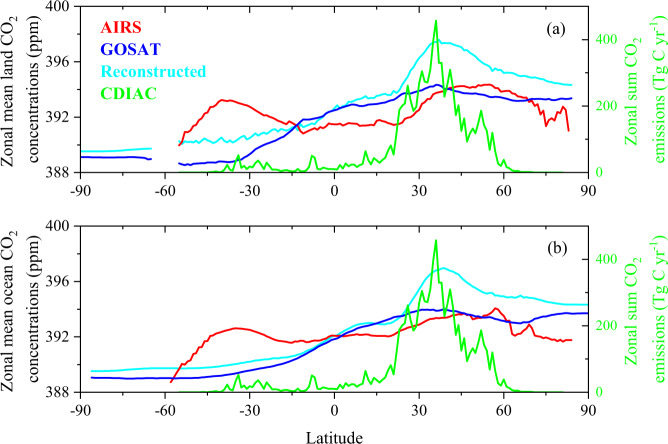
Zonal mean CO_2_ concentrations (ppm) averaged over land (**a**) and over ocean (**b**) for the AIRS, the GOSAT, and the reconstructed data during 2010 to 2013. Zonal sum fossil-fuel CO_2_ emissions (Tg C yr^−1^) are also showed using the right y axis in each panel from Carbon Dioxide Information Analysis Center (CDIAC) averaged during 2010 to 2013.

Figure 10 shows the zonal mean CO_2_ concentrations (ppm) for the AIRS, the GOSAT, and the reconstructed data during 2010 to 2013 averaged over land and averaged over ocean, separately. The zonal mean CO_2_ concentrations for the reconstructed data averaged over land and over ocean both have a similar distribution pattern with the surface CO_2_ concentrations in GOSAT, with higher CO_2_ values in the Northern Hemisphere than that in the Southern Hemisphere, though there are some overestimates in the middle latitudes for the reconstructed CO_2_ concentrations, which is consistent with the high CO_2_ emissions in the middle latitude bands (Fig. 10). In the low and middle latitudes of the Southern Hemisphere, the reconstructed CO_2_ concentrations over land and over ocean are both between the AIRS and the GOSAT range of CO_2_ concentrations, respectively (Fig. 10). We also note that our historical CO_2_ concentrations distributions should be regarded as highly uncertain. However, some plausibility of the CO_2_ concentrations distributions is obtained by comparison with satellite observations (e.g., ARIS, GOSAT satellite CO_2_ concentrations products) at the zonal mean and grid scales.

## Usage Notes

This data is intended for use as a prior in global climate modeling, potential socio-economic implications and mitigation of climate change, and adaptation practices. The historical global monthly distributions of atmospheric CO_2_ concentrations with 1° resolution from 1850 to 2013, including 1 file NetCDF format file named CO2_1deg_month_1850-2013.nc And the spatial distributions of nonuniform CO_2_ under SSP-RCP scenarios are from 2015 to 2150, including 8 files NetCDF format with the naming convention CO2_SSP{XYY}_2015_2150.nc, where X and YY are the shared socioeconomic pathway and radiative forcing level at 2100, respectively. Each NetCDF file contains a monthly variable representing mole fraction of carbon dioxide in air (ppm). including 3 dimensions: time (month of the year expressed as days since the first day of 1850, n = 1968 and 1632 for the historical and the future, respectively); latitude (Degrees North of the equator [cell centres], n = 180); longitude (Degrees East of the Prime Meridian [cell centres], n = 360). We anticipate that the dataset will be widely used by Earth system modeling, agriculture management, and socio-economic analysis, to assess the climate, environmental and socio-economic implications of considering past and on-going inhomogeneous CO_2_ distributions, and for formulating strategies of spatial, as well as global carbon reduction.

## Data Availability

The code used to perform all steps described here and shown in Fig. 1 is contained within a.zip archive named “Code.zip”. The code can be accessed via the Zenodo data repository^23^ (10.5281/zenodo.5021361).
